# A VLP for validation of the *Plasmodium falciparum* circumsporozoite protein junctional epitope for vaccine development

**DOI:** 10.1038/s41541-021-00302-x

**Published:** 2021-04-01

**Authors:** Erwan Atcheson, Adrian V. S. Hill, Arturo Reyes-Sandoval

**Affiliations:** 1 The Jenner Institute, University of Oxford, Oxford, UK; 2 Instituto Politécnico Nacional, IPN. Av. Luis Enrique Erro s/n. Unidad Adolfo López Mateos, Mexico City, Mexico

**Keywords:** Peptide vaccines, Diseases, Infectious diseases, Immunology, Vaccines

## Abstract

Malaria continues to be a pressing global health issue, causing nearly half a million deaths per year. An effective malaria vaccine could radically improve our ability to control and eliminate this pathogen. The most advanced malaria vaccine, RTS,S, confers only 30% protective efficacy under field conditions, and hence the search continues for improved vaccines. New antigens and formulations are always first developed at a pre-clinical level. This paper describes the development of a platform to supplement existing tools of pre-clinical malaria vaccine development, by displaying linear peptides on a virus-like particle (VLP). Peptides from PfCSP, particularly from outside the normal target of neutralizing antibodies, the central NANP repeat region, are screened for evidence of protective efficacy. One peptide, recently identified as a target of potent neutralizing antibodies and lying at the junction between the N-terminal domain and the central repeat region of PfCSP, is found to confer protective efficacy against malaria sporozoite challenge in mice when presented on the Qβ VLP. The platform is also used to explore the effects of increasing numbers of NANP unit repeats, and including a universal CD4^+^ T-cell epitope from tetanus toxin, on immunogenicity and protective efficacy. The VLP-peptide platform is shown to be of use in screening malaria peptides for protective efficacy and answering basic vaccinology questions in a pre-clinical setting.

## Introduction

The need for an effective malaria vaccine is pressing, with almost half a million deaths and 148–304 million clinical cases every year caused by this parasite^1^. The leading malaria vaccine, RTS,S represents a major milestone in malaria vaccine development. However, it is only about 30% efficacious under field conditions^2^, which (at $5–10 per dose) will make it less cost-effective at preventing severe malaria than long-lasting insecticide-treated bednets^3^.

A peptide-based vaccine was amongst the first malaria vaccines to reach clinical study^4^. It consisted of three copies of the NANP tetramer motif from the central repeat region of the major malaria sporozoite antigen, circumsporozoite protein (CSP). This region, and the NANP motif, in particular, had been shown to be a target of neutralising antibodies^5,6^. Despite showing some protective efficacy in a clinical trial, however, it became clear that a construct fusing the repeat region (R) to the Hepatitis B surface antigen (S) was much more immunogenic^7^. When the C-terminal domain of PfCSP, containing a helper T-cell epitope, was included (T), this became the RTS,S vaccine^8^, the only malaria vaccine, or indeed parasite vaccine, to reach Phase III clinical trials^2^. RTS,S is currently undergoing limited pilot distribution in Ghana, Kenya, and Malawi (WHO press release, 24 April 2017).

Although the immunogenicity of short peptides is enhanced by coupling them to virus-like particles (VLPs)^9^, the aim of this paper is not necessarily to advocate VLP-peptide vaccines for clinical use, nor is the aim to obtain high levels of protective efficacy against challenge; one of the main metrics of vaccine efficacy used here is a delay in time to reach 1% blood-stage parasitaemia, previously validated^10,11^ and in fact a more sensitive metric to use when testing the hypothesis that combining vaccines enhances efficacy since sterile protection represents saturation^12^. Rather, the focus of this paper is to explore the potential of VLP-peptide vaccines in pre-clinical screening of epitopes for potential protective efficacy and to answer other basic questions useful to malaria vaccine development. This platform, chemically coupling synthetic peptides via a terminal cysteine residue to the Qβ VLP derived from the Qβ bacteriophage^13^, is tractable and easy to use, allowing novel vaccines to be quickly and inexpensively produced for use in murine malaria vaccine studies.

The utility of this platform in achieving these ends is here demonstrated by the discovery of a linear peptide derived from PfCSP which, when coupled to the VLP Qβ, generates neutralising antibodies capable of protecting mice against challenge with transgenic malaria parasites encoding *P. falciparum* CSP (PfCSP). This finding is given greater significance by the recent identification of neutralising monoclonal antibodies, generated by individuals receiving a radiation attenuated sporozoite vaccine, which recognise this sequence^14,15^; but previous attempts to generate such antibodies in mice by vaccination have not succeeded until now. This peptide sequence lies between the central repeat region and the N-terminal domain of PfCSP and is not included in the RTS,S vaccine.

The Qβ-peptide platform is also used to screen linear epitopes recognised by another recently identified neutralising antibody^16^, spanning the highly conserved “Region I” sequence of the N-terminal domain of PfCSP. These peptides, coupled to Qβ, are highly immunogenic and the antibodies so generated can recognise native CSP; but they confer no protective efficacy on vaccinated mice. This is of use in demonstrating that more needs done to exploit this epitope in a successful vaccination strategy.

Finally, the Qβ-peptide platform is used to determine the effects of increasing the number of NANP repeats per peptide, and of fusing a CD4^+^ T-cell epitope from tetanus toxin to a NANP repeat peptide. Increasing the number of NANP repeats improves immunogenicity and protective efficacy, and including the tetanus, toxin epitope improves anti-NANP antibody affinity, which is shown to be an important correlate of protection.

The discoveries reported here, made using the Qβ-peptide platform, will be of utility in informing strategies to improve the protective efficacy of the RTS,S vaccine or successors.

## Results

### Immunogenicity and protective efficacy of short PfCSP peptides presented on Qβ

**Fig. 1:**
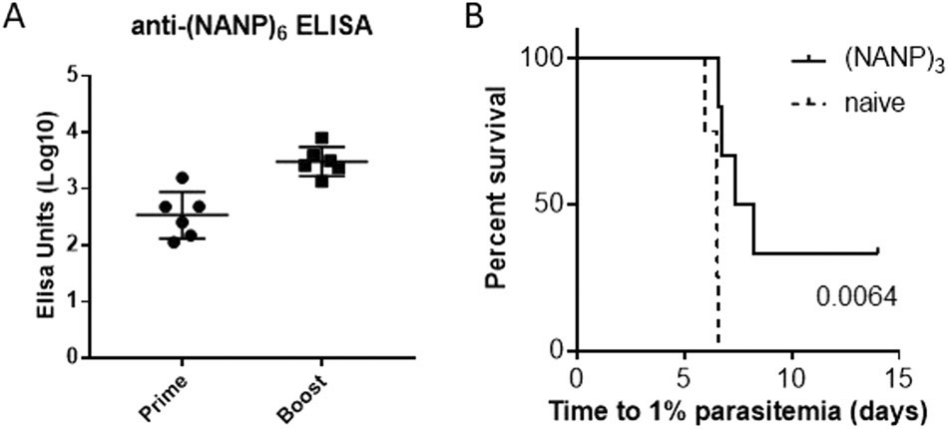
Immunogenicity and protective efficacy of a Qβ-(NANP)_3_ vaccine. BALB/c mice (*n* = 6) per group were vaccinated by intramuscular injection (20 µg per dose, two shots, 3-week prime-boost interval) with (NANP)_3_ chemically coupled to Qβ virus-like particle and delivered with Matrix-M^™^ adjuvant. **A** Standard curve ELISAs performed to determine the antibody response against (NANP)_6_ peptide. Means are shown ± SD. **B** Mice were challenged by intravenous injection of 1000 PfCSP-replacement *P. berghei* sporozoites. The time to reach 1% blood-stage parasitaemia was calculated by linear regression from daily thin blood smears. *P* values from Log-rank tests in comparison to naïves are shown.

When coupled to Qβ, a peptide representing three repeats of the NANP sequence from the central repeat region of PfCSP generates high-titre antibodies (Fig. 1A) and partially protects BALB/c mice from challenge with transgenic PfCSP *Plasmodium berghei* sporozoites (Fig. 1B).

**Fig. 2:**
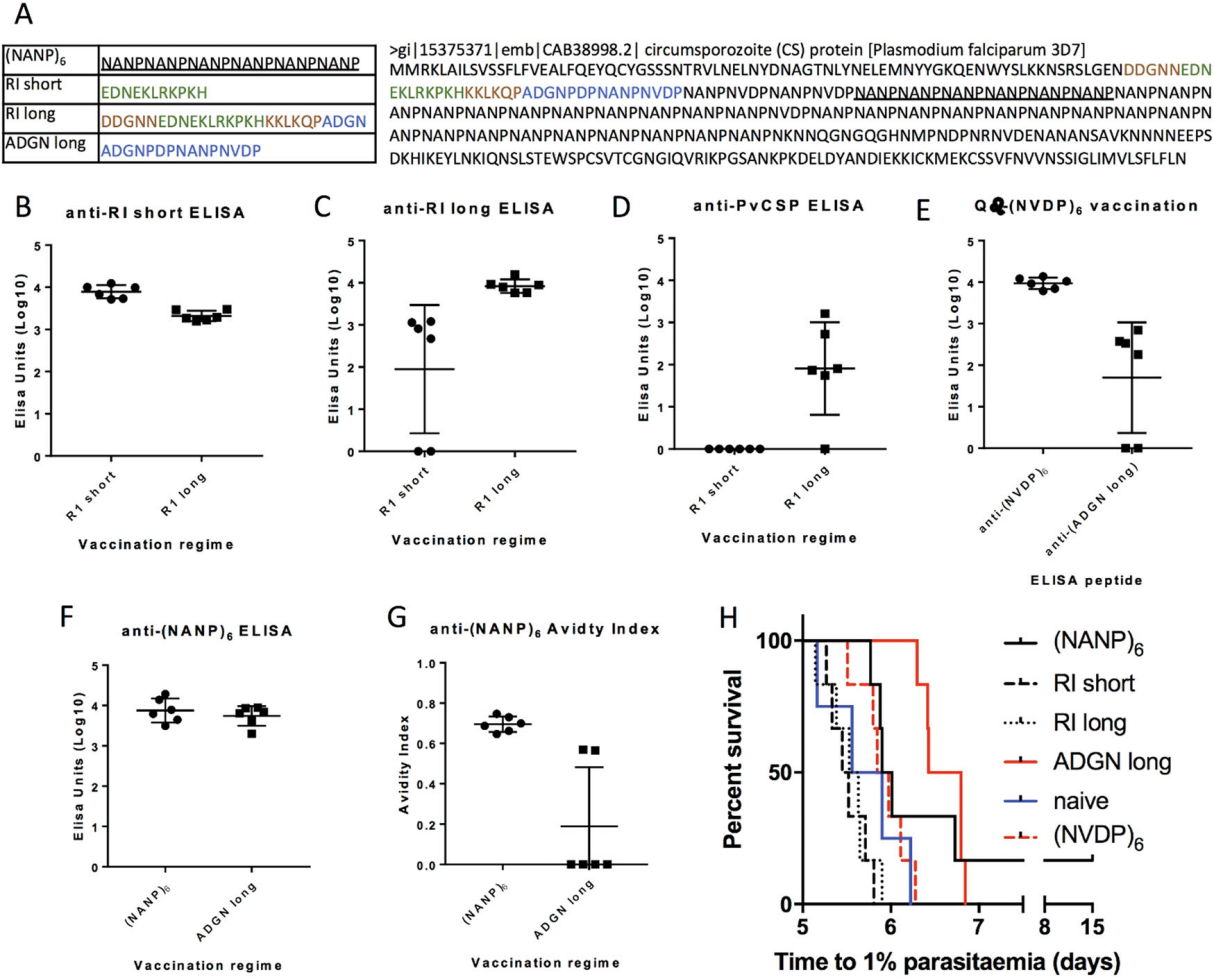
Immunogenicity and protective efficacy of peptides derived from PfCSP delivered as Qβ-peptide vaccines. BALB/c mice (*n* = 5–6 per group) were vaccinated with non-repeat region CSP peptides chemically coupled to Qβ (3 µg per dose, by intramuscular injection, delivered with Matrix-M^™^ adjuvant, three shots using 3-week intervals.) **A** *P. falciparum* CSP peptides used as Qβ-peptide vaccines and location in PfCSP sequence. Standard curve ELISAs were performed using sera taken 2 weeks after the final vaccination, against **B** “RI short” peptide, **C** “RI long” peptide, **D** full-length PvCSP, or **F** (NANP)_6_ peptide; **E** shows mice vaccinated with Qβ-(NVDP)_6_ and ELISAs against peptides shown on the *x*-axis. **G** Avidity index represents the ratio of sera treated with 7 M urea to untreated sera in ELISAs. Means are shown ± SD. Vaccination received shown on *x*-axis except in the case of (**E**). **H** Mice were challenged 3 weeks after the final shot with 1000 transgenic *P. berghei* sporozoites expressing PfCSP, and time to reach 1% blood-stage parasitaemia determined by linear regression using daily thin blood smears.

To see if peptides from other regions of PfCSP could likewise generate protective immunity when administered as Qβ-peptide vaccines, two peptides (“RI short” and “RI long”) was constructed based on the core and expanded linear sequence recognised by a neutralising monoclonal that targets the N-terminal domain of PfCSP in Region I; see Fig. 2A. Despite being highly immunogenic in homologous and heterologous peptide enzyme-linked immunosorbent assays (ELISAs) (Fig. 2B, C), and, in the case of RI-long, showing cross-species reactivity against full-length PvCSP-210 (Fig. 2D), neither of these Region I-based Qβ-peptide vaccines conferred any protection against challenge with transgenic PfCSP *P. berghei* sporozoites (Fig. 2H). In addition, a Qβ-(NVDP)_6_ vaccine was tested, to see if antibodies against the NVDP tetramer, present in three non-consecutive copies in the PfCSP 3D7 sequence, could neutralise sporozoites, given the protective efficacy of anti-NANP antibodies. Despite generating antibodies against (NVDP)_6_ and a native peptide from PfCSP containing a copy of NVDP (“ADGN long”) (Fig. 2E), the Qβ-(NVDP)_6_ vaccine showed no protective efficacy (Fig. 2H). The lack of efficacy of these three vaccines demonstrates that any contribution to protective efficacy from the adjuvant, Matrix M, alone, is absent or negligible.

A forth peptide from PfCSP, “ADGN long”, lying adjacent to Region I at the junction between the N-terminal domain and the repeat region of PfCSP, (see Fig. 2A for sequence and position) was likewise chemically coupled to Qβ and administered to mice. Qβ-(ADGN long) was found to be both highly immunogenic (Fig. 2F, G) and partially protected mice against challenge with transgenic PfCSP *P. berghei* sporozoites (Fig. 2H).

Qβ-(ADGN long) contains a single NANP tetramer within its sequence, and generates anti-NANP antibodies (Fig. 2F). These are, however, of extremely low affinity (Fig. 2G). This suggested that these anti-NANP antibodies were not principally responsible for mediating the protective efficacy of the Qβ-(ADGN long) vaccine.

**Fig. 3:**
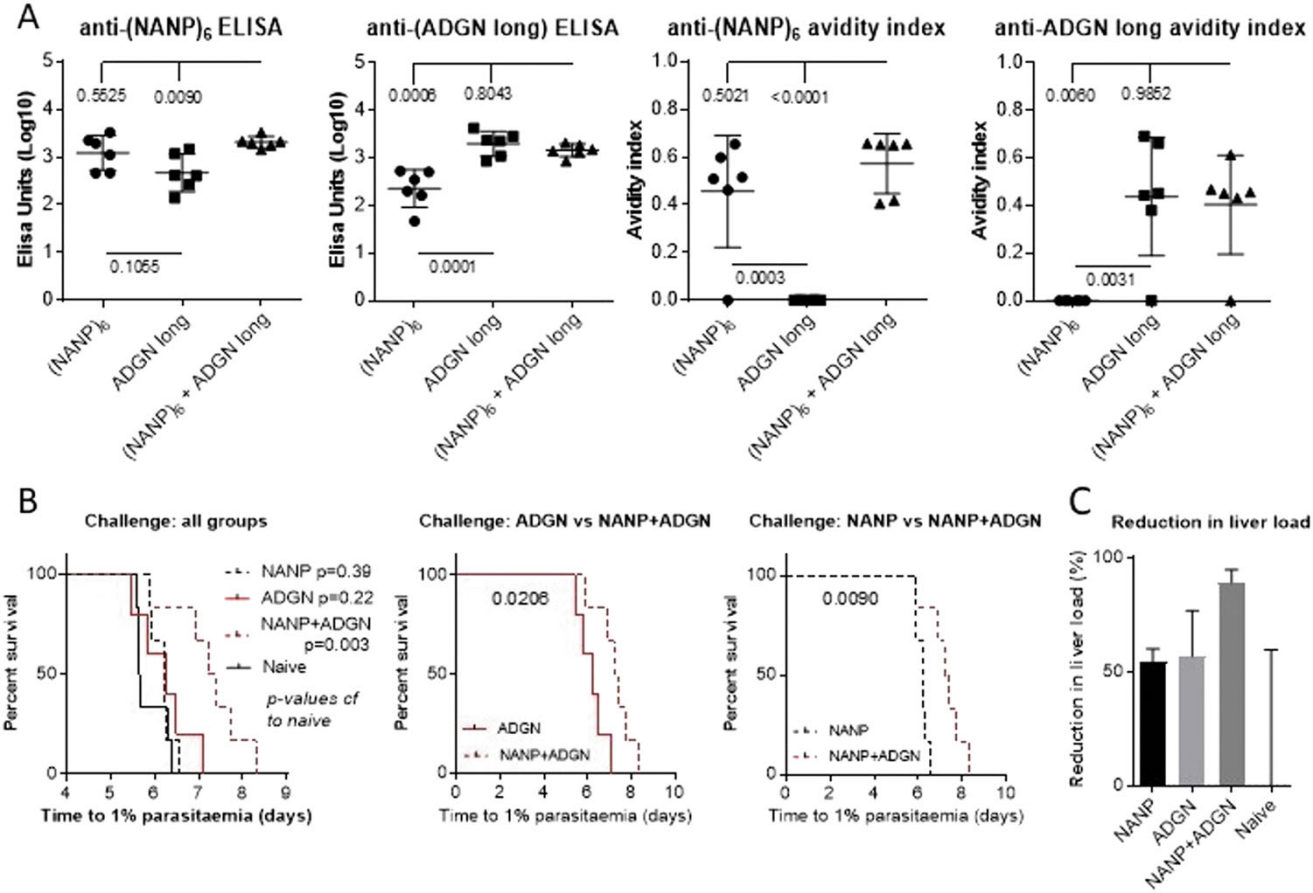
Effects on immunogenicity and protective efficacy of combining Qβ-(NANP)_6_ and Qβ-(ADGN long) vaccines. BALB/c mice (*n* = 6 per group) were vaccinated with two shots by intramuscular injection of 1 µg of each Qβ-peptide in Matrix-M^™^ adjuvant into separate legs using a 3-week prime-boost interval. **A** Standard curve ELISAs from sera taken 2 weeks post-boost, and avidity. Numbers represent *p* values from ANOVA with Bonferroni’s multiple comparisons test. **B** Mice were challenged 3 weeks post-boost by intravenous injection of 1000 PfCSP replacement *P. berghei* sporozoites into the tail vein. The time to reach 1% blood-stage parasitaemia was calculated by linear regression from daily thin blood smears. Numbers represent p-values from log-rank tests between indicated groups. Means are shown ± SD. **C** Reduction in liver load, the ratio of infected hepatocytes in vaccinated (*L*_*V*_) compared to naïve mice (*L*_*N*_), was calculated using the formula Reduction (%) = (1 − *i*_*V*_)*100, where the vaccine-reduced infectivity *i*_*V*_ = *L*_*V*_/*L*_*N*_ = *g*^*Tn−Tv*^, where *g* is the blood-stage growth rate, *T*_*N*_ the median time-to-1% in naïve mice, and *T*_*V*_ the time-to-1% in vaccinated mice.

In theory, two vaccines generating immune responses against different targets will, if either is weakly protective alone, show markedly enhanced efficacy when combined^12^. To test whether “ADGN long” represents a truly novel epitope, rather than its effects being mediated through anti-NANP antibodies as with Qβ-(NANP)_6_, an experiment was carried out combining Qβ-(ADGN long) and Qβ-(NANP)_6_ vaccines under suboptimal conditions, with dosage reduced compared to previous experiments (Fig. 1) to allow maximum sensitivity in detecting any enhancement in protective efficacy. Enhanced protective efficacy was obtained with the combination of Qβ-(NANP)_6_ and Qβ-(ADGN long) compared with either subunit vaccine alone (Fig. 3B). Given that the anti-(NANP)_6_ and anti-(ADGN long) titres and avidity indices were the same in the single-component and combination groups (Fig. 3A), the only remaining explanation for enhanced efficacy is that these vaccines elicit immune responses against different epitopes.

To further test whether the enhancement in protective efficacy was consistent with theoretical predictions, a mathematical model developed by our group^12^ was adapted to present purposes. From that mathematical model, the following expression can be derived

1 $$\frac{{{{L}}_{{V}}}}{{{{L}}_{{N}}}} = i_{V1} \ast i_{V2} = g^{Tn - Tv}$$

where *L*_*V*_ and *L*_*N*_ are the numbers of infected hepatocytes in vaccinated and naïve mice, respectively; *i*_*V*1_ and *i*_*V*2_ are the reductions in infectivity caused by two different immune components (in this case, against (NANP)_6_ and ADGN-long); *g* is the blood-stage growth rate (calculated in ref. ^12^ to be *g* = 3.5); and *T*_*N*_ and *T*_*V*_ are the time-to-1% values of the naïve and vaccinated mice, respectively. The reduction in liver load for each group compared to naïve mice can be calculated as 100*(1 − *i*_*V*1_*i_*V*2_) and is shown graphically in Fig. 3C, where median values are used for the calculations as the distribution of *T* is theoretically skewed^12^; this can be seen in the present data where the time-to-1% values in challenged naïve mice fall in a normal range of 5.6–6.4 d (Fig. 3B) but with the two mice showing time-to-1% of 6.4 d showing extrapolated liver burdens 50% less than the four mice showing time-to-1% of 5.6 d. The time-to-1% was also half a day longer than in the previous experiment (Fig. 2) explaining the lack of efficacy of Qβ-(NANP)_6_ alone (Fig. 3). A predicted value for the reduction in liver load in the combination NANP + ADGN group can be calculated from the actual values of the Qβ-(NANP)_6_ and Qβ-(ADGN long)-only groups, which have reductions in a liver load of 54% and 57%, respectively. The predicted reduction in liver load from the combination NANP + ADGN group is 80%, and the actual reduction is 89%. A Wilcoxon signed-rank test comparing the predicted and actual reduction confirms the values to be very close, with a *p* value for discrepancy of 0.44.

In summary, the Qβ-peptide platform enabled the discovery of a neutralising PfCSP epitope. The utility of the platform was further demonstrated by investigating the effect of increasing the number of (NANP) units per peptide.

### Increasing the number of units repeats in Qβ-(NANP) vaccines

**Fig. 4:**
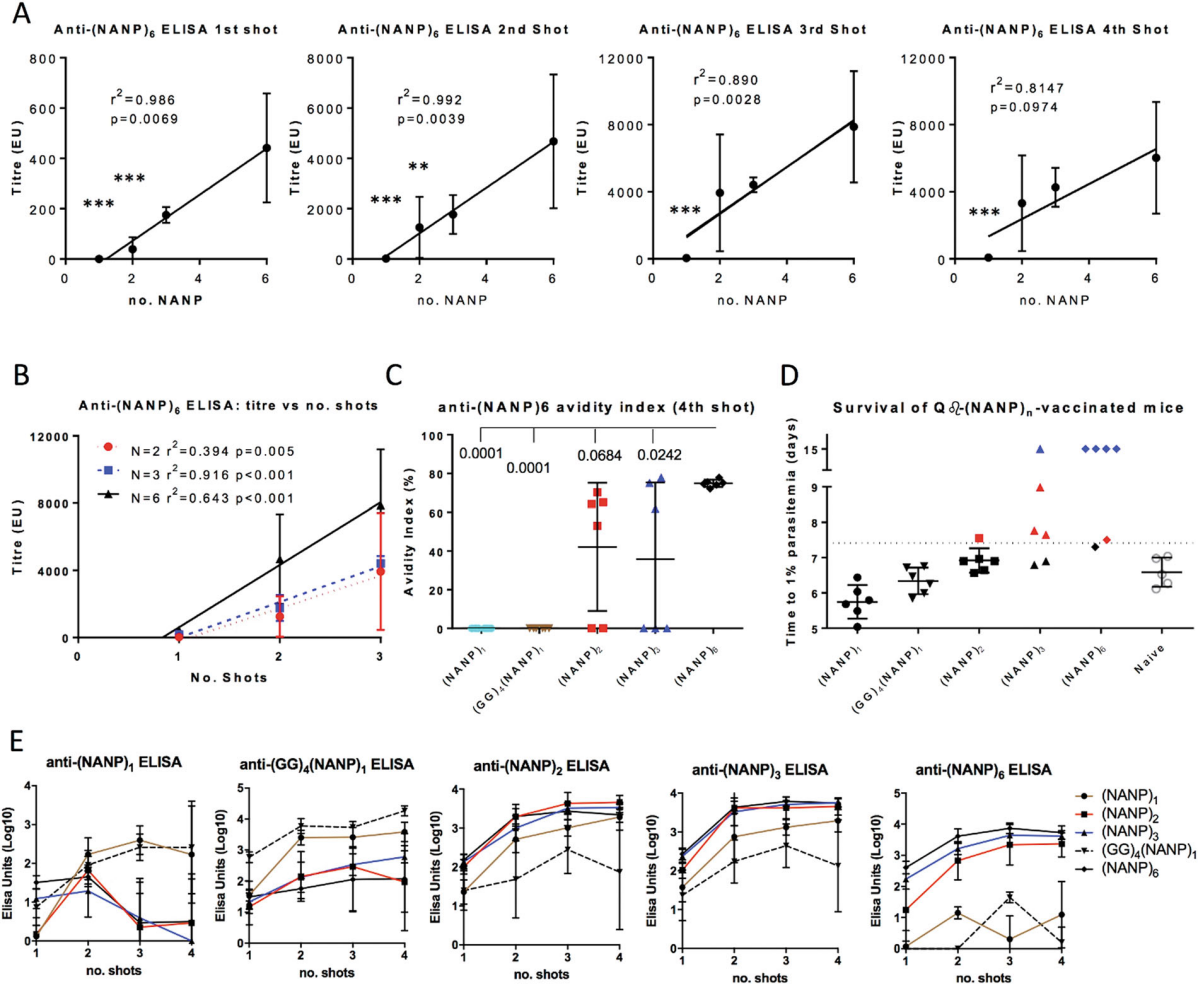
Effects on immunogenicity and protective efficacy of increasing the number of units repeats in Qβ-(NANP) vaccines. Qβ-(NANP)_*n*_ VLPs were constructed by synthesising peptides with varying numbers of (NANP) unit repeats (no. NANP = 1, 2, 3, 6) and (GG)_4_(NANP)_1_ and chemically coupling them to Qβ. These VLPs were used to vaccinate BALB/c mice (*n* = 6 per group) by intramuscular injection of 3 µg doses in Matrix-M^™^ adjuvant, with four shots given at 3-week intervals. **A** Serum was taken 2 weeks after each shot and used in standard curve ELISAs against (NANP)_6_. *r*^2^ and *p* values from linear regressions are shown. Asterix represents significance levels of *p* < 0.01 (**) and *p* < 0.001 (***) by ANOVA in comparison to Qβ-(NANP)_6_. **B** Titre versus a number of shots for each Qb-(NANP)_*n*_ group, with *N* = 2, *N* = 3 or *N* = 6 as shown. **C** The avidity indices 2 weeks post-fourth shot; shown are *p* values from ANOVA with Bonferroni’s multiple comparisons test in comparison to Qβ-(NANP)_6_-vaccinated mice. **D** Three weeks after the fourth vaccination mice were challenged by intravenous injection of 1000 PfCSP transgenic *P. berghei* sporozoites. Thin blood smears were taken daily to determine the time to reach 1% blood-stage parasitaemia by linear regression. In challenge data, points coloured blue represent sterile protection, and points coloured red to represent a delay in time-to-1%, defined as time-to-1% greater than average naïve time-to-1% plus 2 standard deviations, with the boundary represented by a dotted line. **E** Sera from mice in (**A**) were also used to determine antibody titres specific for each variable number of (NANP) repeats, 2 weeks post-vaccination; graph titles represent the peptide used to coat ELISA plates, and lines representing vaccination groups as shown. Means are shown ± SD.

Peptides consisting of 1, 2, 3 or 6 NANP repeats were synthesised and coupled to Qβ to see if increasing the number of NANP repeats would increase the immunogenicity of Qβ-(NANP) vaccines. ELISAs performed on sera from BALB/c mice vaccinated with these Qβ-(NANP)_*n*_ vaccines demonstrated an apparently linear relationship between titre and number of NANP repeats (Fig. 4A), at least for the first two shots (first shot, *r*^2^ = 0.986, *p* = 0.007; second shot, *r*^2^ = 0.992, *p* = 0.0039). The relation still existed, though more loosely, after a third vaccination (*r*^2^ = 0.890, *p* = 0.0028) but a failure to boost titres in most mice after a fourth shot seems to have terminated the relationship (*r*^2^ = 0.815, *p* = 0.097). Excluding the non-boosting fourth shot, the relationship between a number of shots and titre also appears to be linear for Qβ-(NANP)_2_ (*r*^2^ = 0.394, *p* = 0.005), Qβ-(NANP)_3_ (*r*^2^ = 0.916, *p* < 0.0001) and Qβ-(NANP)_6_ (*r*^2^ = 0.643, *p* < 0.0001), though the linearity of this relationship is perhaps only convincing for Qβ-(NANP)_3_ (Fig. 4B). The avidity index was also highest for mice vaccinated with Qβ-(NANP)_6_ (Fig. 4C), and higher titres and affinity translated into increasing protection with increasing numbers of NANP repeats (Fig. 4D).

Qβ-(NANP)_1_- and Qβ-(GG)_4_(NANP)_1_-vaccinated mice, though generating no or low responses against (NANP)_6_ in ELISAs, did generate high titres against (NANP)_1_ and (GG)_4_(NANP)_1_ peptide as ELISA coating antigens; indeed, they generated higher titres against those peptides than any Qβ-(NANP)_*n*_ with two or more NANP repeats (Fig. 4E). Similarly, Qβ-(NANP)_2_-vaccination generated the highest titres in an anti-(NANP)_2_ ELISA. In anti-(NANP)_3_ ELISAs Qβ-(NANP)_6_ mice produced the highest titres, as with (NANP)_6_ ELISAs, suggesting that (NANP)_*n*_ peptides of one or two repeats have a different conformation from that of three or more.

Theoretically, the relationship between the number of NANP repeats and anti-(NANP)_6_ titre is not expected to remain linear indefinitely. The point at which the relationship saturates is yet to be experimentally determined, but the mathematical model developed below suggests that it will saturate at some point. The model, relating titre to dose, *V*, a number of unit repeats, *N*, and density of peptides, *∂*, on each VLP, derives from the following assumptions:

1. There is a fixed and finite quantity of epitope-specific B-cells in a naïve mouse, equal to *B*_max_.
2. The net quantity of antibodies *R* secreted by a given activated epitope-specific B-cell *B*_*A*_ and descendent B-cells resulting from its proliferation will be a constant: *R* = f * B_*A*_, where *f* is a function describing the number of B-cells generated by the proliferation of the original activated B-cell *B*_*A*_ and the number of antibodies released by each.
3. The probability that a given epitope on a given VLP binds and activates a B-cell after a VLP/B-cell encounter, *p*_*n*_, is independent of *∂*, *V*, and *N*.
4. The probability *p*_*a*_ that a given B-cell is activated is independent of the activation status of any other B-cells.

If ∂, *V* and *N* are given, then the number of activated B-cells *B*_*A*_ is

2 $$B_A = B_{\rm{max}} \ast p_a$$

Since *R* = f * B_*A*_, the maximum number of antibodies released *R*_max_ = f * B_max_. Therefore

3 $$R = R_{\rm{max}} \ast p_a$$

Failure to activate a B-cell, *p*_*−a*_, occurs when each unit epitope on each VLP which encounters a B-cell fails to trigger activation. A given unit epitope on a VLP has a probability *p*_*n*_ of activating the B-cell, and probability *p*_*−n*_ = 1 − *p*_*n*_ of failing to activate the B-cell. On a given VLP there are ∂*N unit epitopes; the probability that they all fail to activate the B-cell is (1 − *p*_*n*_)^∂*N^. If *p*_*V*_ is the probability that a VLP encounters a B-cell, then on average a B-cell will have *p*_V_*V encounters. Thus the probability that each unit epitope on each VLP which encounters a B-cell fails to activate it is *p*_−__*a*_ = (1 − *p*_*n*_)^∂*N*pV*V^. If the dose *V* is expressed in arbitrary units (e.g. µg or ng) then *p*_*V*_ can for practical purposes be set equal to one. Thus

4 $$p_a = 1 - p_{ - a} = 1 - \left( {1 - p_n} \right)^{\partial \ast N \ast V}$$

and from (3) and (4)

5 $$R = R_{\rm{max}} \ast \left( {1 - \left( {1 - p_n} \right)^{\partial \ast N \ast V}} \right)$$

Equation (5) suggests that the relationship will only be linear at small scales, and should in fact be saturating. This is found to be the case with an experiment in mice comparing two Hepatitis B surface antigen-based VLPs, R21, and RTS,S, which differ only in the density of CSP antigen per VLP; differences in the immune profile between the two VLPs fit very closely the predicted difference from the model above (Adrian Hill, pers. comm).

Thus, increasing the number of NANP tetramers per peptide improves immunogenicity and protective efficacy, though in theory with diminishing returns. Next, the platform was used to investigate whether including a short CD4^+^ T-cell epitope from tetanus toxin would improve the affinity and protective efficacy of Qβ-(NANP)-based vaccines.

### Effects on immunogenicity and protective efficacy of fusing a tetanus toxin CD4^+^ T-cell epitope with (NANP)_3_

**Fig. 5:**
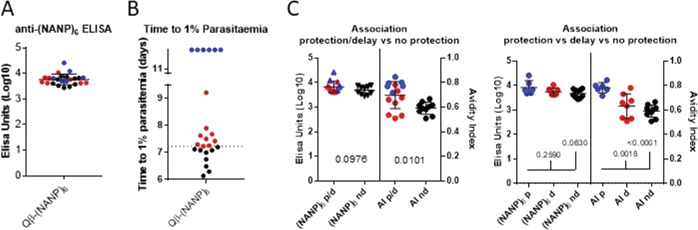
Avidity index as a correlate of protection in Qβ-(NANP)_6_-vaccinated mice. BALB/c mice (*n* = 24) were vaccinated with Qβ-(NANP)_6_ by intramuscular injection, using 1 µg per dose, two shots, a 3-week prime-boost interval, and Matrix-M^™^ adjuvant. **A** Serum was collected from mice 2 weeks post-boost and standard curve ELISA performed against (NANP)_6_. **B** Mice were challenged intravenously with 1000 PfCSP transgenic *P. berghei* sporozoites 3 weeks post-boost. Daily thin blood smears were used to determine the time to reach 1% blood-stage parasitaemia by linear regression. Mice with sterile protection are coloured blue, and those with a delay in time to reach 1% blood-stage parasitaemia coloured red, where “delay” is defined as time-to-1% greater than the mean of naïve challenged mice plus two standard deviations. **C** Associations with protection were obtained by categorising mice into two or three groups following challenge: protected or delayed (p/d) vs. no delay (nd), or protection (p) vs. delay (d) vs. no delay (nd). Numbers represent *p* values *t* tests (comparing two groups) or ANOVA with Bonferroni’s multiple comparisons test (comparing three groups). Means are shown ± SD.

Despite similar anti-(NANP)_6_ titres (Fig. 5A), mice vaccinated with Qβ-(NANP)_6_ show variable outcomes following challenge (Fig. 5B). When titres are very similar, the affinity of anti-(NANP)_6_ antibodies associates strongly with the outcome, when mice are divided into two groups (protected or showing delay in time to reach 1% parasitaemia, vs. no evidence of delay) or three groups, as shown in Fig. 5C.

**Fig. 6:**
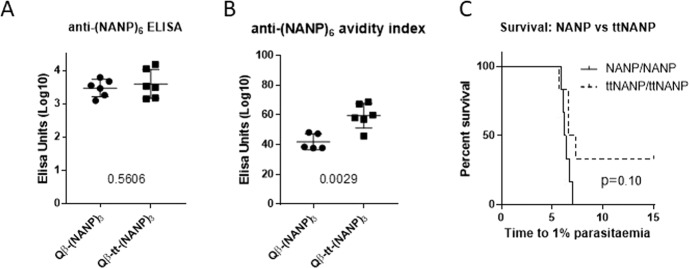
Effects on immunogenicity and protective efficacy of tetanus toxin CD4^+^ T-cell epitope fusion with Qβ-(NANP)_3_. Qβ VLPs were chemically coupled with tetanus toxin P2 epitope fused to (NANP)_3_ (Qβ-tt-(NANP)_3_) or just to (NANP)_3_ (Qβ-(NANP)_3_) and used to vaccinate BALB/c mice (*n* = 6 per group) by intramuscular injection, using 1 µg per dose, two shots, a 3 week prime-boost interval, with Matrix-M^™^ adjuvant. **A** Serum was collected from mice 2 weeks post-boost and standard curve ELISAs performed against (NANP)_6_. **B** The avidity index was calculated by taking the ratio of anti-(NANP)_6_ titres with and without treatment of sera with 7 M urea. Numbers on graphs represent *p* values from *t* tests between groups. **C** Mice were challenged intravenously with 1000 PfCSP transgenic *P. berghei* sporozoites 3 weeks post-boost. Daily thin blood smears were used to determine the time to reach 1% blood-stage parasitaemia by linear regression. *p* Value from log-rank test. Means are shown ± SD.

For this reason, the P2 universal T-cell epitope from tetanus toxin (tt) was fused to (NANP)_3_ to see if this would improve the affinity and hence the protective efficacy of (NANP)-based vaccines. Figure 6A shows a marginal improvement in titre (*p* = 0.56, Fig. 6A), but a highly significant increase in the avidity index of anti-(NANP)_6_ antibody (*p* = 0.0029, Fig. 6B) resulting from the inclusion of the tt epitope with (NANP)_3_, compared to (NANP)_3_ alone, presented on Qβ. Improved protective efficacy was seen with Qβ-tt-(NANP)_3_ vaccination compared with Qβ-(NANP)_3_, but this was not significant (*p* = 0.1, Fig. 6C). Thus, adding a universal CD4^+^ epitope to a NANP-based vaccine can improve antibody affinity, and thus potentially increase protective efficacy.

## Discussion

The VLP-peptide platform is a simple and straightforward way of answering important questions in vaccine development, having been used here to identify a target of neutralising antibodies in PfCSP, establish that increasing numbers of the NANP unit repeat improves immunogenicity and protective efficacy, and that inclusion of a CD4^+^ T-cell epitope can improve the affinity of anti-(NANP) antibodies.

The finding that protective immunity against malaria can be generated by Qβ-(ADGN long) vaccination of mice is particularly exciting, as this junctional epitope, situated between RI in the N-terminal domain and the central repeat region of PfCSP, has been recently identified as a target of potently neutralising dual-specificity antibodies generated in humans following irradiated sporozoite (PfSPZ) vaccination^14,15^. Such antibodies recognise both the junctional epitope and the central repeat region; Qβ-(ADGN long) also elicits antibodies against both regions, and it would be interesting to find out if these antibodies are likewise of dual specificity. Vaccination of BALB/c mice by conjugation of KQPADGNPDPNANPNVDPN to keyhole limpit haemocyanin (KLH) carrier protein elicited only non-neutralising antibodies^15^. The success of our vaccination regime is likely due to the use of a VLP, Qβ, to present the junctional peptide, as VLPs elicit higher titres of the antibody with higher affinity. If this is the reason for the difference in outcomes, it underscores the utility of the VLP-peptide platform to screen epitopes for protective efficacy, as carrier proteins such as KLH, although inducing antibody responses to peptides, are probably not sufficiently immunogenic to raise high-quality, high-titre antibodies. However, it is possible that the addition of ‘KQP’ at the beginning of the above sequence abrogates the production of neutralising antibodies. It will be important to settle this question, as the most obvious application of these findings is to warrant an extension of the existing RTS,S and R21^17^ vaccines to include the junctional epitope. It has been shown here that combining vaccines including (NANP)_6_ and the junctional epitope ADGN-long enhances protective efficacy compared to vaccines containing either epitope alone. The efficacy of either vaccine was low, but the purpose of the present study was only to identify the potential of a novel epitope in comparison with a known target of neutralising antibodies, NANP. The exact limits of the optimal extension of ADGN-long could be determined pre-clinically, using a VLP-peptide platform as here.

RI-based Qβ-peptide showed no efficacy, despite the presented epitopes being recognised by a potently neutralising antibody^16^, and despite the antibodies generated by Qβ-RI vaccines showing excellent recognition of full-length PvCSP, suggesting that if a vaccine targeting RI could be made to work, it could have cross-species efficacy. The best results obtained with Region I or II peptide-based vaccines have used longer versions than those used in this paper, but still only achieved 50-60% protection against a very low dose challenge (100 sporozoites)^18^, or reduced liver burden by 18%, low compared to 98% reduction obtained with a repeat-region based vaccine in the same study^19^. The epitope is cryptic, not being recognised when presented within the full-length CSP^20^, but inconsistently, so, being elicited in some volunteers by vaccination with radiation attenuated sporozoites^14,15^. Separate vaccination with the N-terminal region may succeed in eliciting neutralising antibodies, but that strategy would probably increase the expense of the vaccine if combined with, say, RTS,S or R21. A single-component vaccine consisting of the repeat region and the N-terminal domain only, and missing the C-terminal domain, might allow neutralising antibodies to be generated against Region I as then CSP will be in a potentially more immunogenic conformation^21^.

The Qβ-peptide platform was also used to uncover an apparently linear relationship between the number of NANP repeats and anti-NANP titres. It has previously been shown that a PfCSP vaccine with 19 NANP repeats was more immunogenic and protective than one with 5 repeats^22^. This was shown again here with short NANP peptides on Qβ with 1, 2, 3 and 6 NANP repeats, which showed increasing immunogenicity and protective efficacy with the number of repeats. The apparently linear relationship has not previously been observed, for no experiment has used more than two alternate NANP repeat numbers. The mathematical model here-in has presented, relating titre to peptide density, number of unit repeats per peptide, and dose, predicts a saturating effect, with extra NANP repeats ultimately failing to significantly increase titres. Increased density of NANP on a VLP has previously been associated with improved immunogenicity (675) and indeed antigen density also appears to dictate immune responses to blood antigens (676) The model accurately fits data from R21 and RTS,S varying dose and density (Florian Brod, personal communication) but it remains to be seen whether it also holds true when the number of unit repeats is increased. Potentially confounding this is evidence (Fig. 4E) that, at least at low numbers of (NANP) repeats, conformation varies; an assumption of the model is that this does not happen. If the model holds for NANP repeats, then the main benefit of longer NANP sequences presented at higher densities on VLPs will be dose-sparing, helping reduce the cost of the vaccine, and hence increase its cost-effectiveness.

If short-peptide VLPs displaying malaria B-cell epitopes are to play some role in future vaccine development, it would be useful to know how to optimise immune responses against them. Malarial helper T-cell epitopes, or those from unrelated species such as the universal T-cell epitope from tetanus toxin, P2, have been used with most MAPs displaying CSP repeat peptides^23–33^, because without T-cell help the immune responses are very weak or absent^24,25^. With the Qβ-peptide vaccines used in this thesis, this is evidently not an issue, as high titres were always achieved against the peptide presented on the VLP. Nevertheless, a fusion of the P2 epitope to (NANP)_3_ for presentation on Qβ enhanced titres, though only slightly and the improvement was not statistically significant. The affinity of anti-NANP titres was, however, significantly improved if P2 was present, which could be of value because this paper also presented data to show that affinity determines challenge outcome when anti-NANP titres are equal. Further studies could look at the effects on the immunogenicity of other T-cell epitopes, and warrants exploring the addition of non-native T-cell epitopes to existing PfCSP VLPs such as RTS,S and R21.

Qβ can elicit CD8^+^ T-cell responses in addition to CD4^+^ and antibody responses, but only at much higher doses than used here^34^. This platform could therefore also be used to explore CD8^+^ T-cell responses in future. As total IgG has been found to correlate better than any given IgG subtype with protection in this model (authors, unpublished data), the contribution to the protection of particular IgG subtypes was not explored here. However, it would be of interest to further examine the mechanism of protection induced by these vaccines by, for instance, cleaving the Fc region of elicited antibodies prior to sporozoite neutralisation assays to determine whether there are Fc-mediated protective mechanisms, or whether protection is mediated entirely by blocking hepatocyte binding and entry. The persistence of antibody titres was not measured in this study, as the platform is not being developed for clinical use but rather as a tractable system for screening and exploring basic questions about the protective efficacy of epitopes. It would be of interest, however, to examine the longevity in this system, as another anti-CSP VLP platform, Rv21, was previously shown to elicit antibodies persisting at very high levels for over 9 months^35^.

In summary, the results here reported establishing that VLP-peptide vaccines are a tractable and inexpensive platform for conducting pre-clinical work important to informing clinical vaccine development.

## Methods

### Vaccination

Vaccinations were performed by intramuscular injection using a 25 G needle of 25 µL vaccine formulation each into the right and left hind leg muscles (unless otherwise stated) of isofluorane-anaesthetised mice. Vaccines were administered at ranges from 1 to 20 µg, typically with two to three vaccinations administered at 3-week intervals unless otherwise stated. Matrix-M^™^ adjuvant (Novavax AB, Uppsala, Sweden) was used at 12 µg per dose. Adjuvants were kindly provided by Dr. Anita Milicic from the Jenner Institute adjuvant bank.

### Mouse strains used

Six-week-old female BALB/c (H-2^d^) mice were used for vaccination experiments, with age-matched controls. To outbred mice and BALB/c mice were used for parasite maintenance and mosquito feeds. All mice from Harlan/Envigo.

### Ethics statement

All animals and procedures were used in accordance with the terms of the United Kingdom Home Office Animals Act Project License. The procedures were approved by the University of Oxford Animal Care and Ethical Review Committee (PPL 30/2889 and P9804B4F1).

### Infection of *Anopheles stephensi* mosquitoes with *P. berghei*

Cryopreserved mouse blood stocks of wild type or transgenic *P. berghei* from liquid nitrogen were defrosted and immediately administered to naïve BALB/c or TO mice by intraperitoneal injection (100 µL). Thin blood smears were taken daily and when gametocytes were observed mice were anaesthetised by intramuscular injection (Rompun/Ketaset) for mosquito feed. Mosquitoes starved for 2 h were allowed to feed for 10–15 m on anaesthetised infected mice. Blood was taken from mice to confirm exflaggelation of gametocytes by microscopy. After feeding, mosquitoes were returned to fructose/P-amino benzoic acid on cotton wool and maintained in the Jenner Institute insectary (19–21 °C, 12 h light/dark cycle). One week after feeding a second feed was performed on an anaesthetised naive mouse to improve sporozoite yields. Mosquitoes were maintained for a total of 21 days prior to dissection of sporozoite-infected salivary glands.

### Dissection of mosquito salivary glands and challenge of mice with sporozoites

Twenty-one days after feeding on *P. berghei* infected mice, mosquitoes were sedated at 4 °C for dissection. Salivary glands were dissected from mosquitoes under a microscope and removed by pipette into a glass tissue homogeniser containing 100 µL Schneider’s insect media with 10% fetal bovine serum. Sporozoites were liberated from salivary glands by gently homogenising three times and counted using a haemocytometer. Sporozoite concentration was adjusted to 10^4^ sporozoites/mL for intravenous injection into the tail vein of mice of 100 µL (1000 sporozoites per dose, by insulin syringe).

### Thin blood smears and calculation of time to reach 1% blood-stage parasitaemia

Daily thin blood smears were prepared on glass slides from a drop of blood taken from the tail tip of challenged mice. Slides were fixed in methanol then stained in 5% Giemsa (Sigma) for 30 min and washed in water. 1000 red blood cells were counted for three to five consecutive days until the mouse reached 1% blood-stage parasitaemia. Time to reach 1% blood-stage parasitaemia was calculated by linear regression of log_10_ percentage parasitaemia against time post-challenge, as described in^11^. Mice without parasites by day 15 were considered to have been conferred sterile protection against challenge. All data from all experiments carried out in this study are provided in the manuscript. Epitopes not showing any evidence of protective efficacy after one challenge experiment with 6 mice were not further pursued. All epitopes demonstrating evidence of protective efficacy were tested in at least two independent challenge experiments.

### Production of transgenic *P. berghei* parasites

*P. berghei* parasites expressing full-length PfCSP 3D7 (as shown in Fig. 2A) in place of PbCSP were produced by inserting an additional copy of the PfCSP gene at the 230 p locus, under the control of the *P. berghei* UIS4 promoter, using the gene insertion/marker out approach^17^.

### Qβ VLP production, purification and chemical coupling

Qβ-transformed *Escherichia coli* from glycerol stock was grown to 1 mL in LB/carbenicillin, then transferred to 1 L M9 media (with 2 mL MgSO_4_, 5 mL 40% glucose, 50 mL casamino acid, 500 µL vitamin B1 and 100 mg/mL carbenicillin) and incubated at 37 °C for 18 h. Cells were pelleted (5000*g*, 25 min, 4 °C) and the supernatant discarded. The pellet was resuspended in PBS, centrifuged again (20 min, 14,000*g*), and the supernatant discarded. The pellet was lysed using lysis buffer (20 mM NaPO_4_ pH 7.5, 0.1% triton x-100, 5 mM EDTA, 100 U/g cells Benzonase, 10 µL/g cells Lysonase, 10 µL/ml protease inhibitor), and freeze/thawing the pellet in dry ice twice. Lysed cells were sonicated for 1 min (15 s on/30 s off, 30% intensity), centrifuged at 14,000*g* for 25 min, and the supernatant collected and filtered. Fractogel purification was carried out using 20 mM NaPO_4_ pH 7.2 buffer with either 150 mM or 1 M NaCl, followed by size exclusion chromatography.

Coupling Qβ peptides were performed by derivatising Qβ with reactive groups using succinimidyl-6-[(β-maleimidopropionamido)hexanoate] (SMPH) at 10× molar excess SMPH (1 h, 50 g RT), followed by three 1 m 100 kDa spin filtrations with PBS (Amicon 0.5 mL) to remove free SMPH. Peptides were synthesised with free cysteines rendering SATA derivation unnecessary. Peptides were incubated with SMPH-derivatised Qβ for 3 h (50 g, RT) and Qβ-VLPs stored at −20 °C. Qβ-VLPs consist of 180 identical subunits with four binding sites each and the binding efficiency of peptides was consistently at 80% for vaccines produced in this study. In consequence, approximately 575 peptides are presented per Qβ-VLP^13^.

A list of peptides coupled to Qβ and used as Qβ-peptide VLPs in this paper is given in Table 1. All peptides were synthesised by ThinkPeptides.

**Table 1:** Peptides coupled to Qβ.

Name	Sequence
(NANP)_3_	CGGNANPNANPNANP
ttNANP	CGGQYIKANSKFIGITENANPNANPNANP
(NANP)_1_	CGGNANP
(GGGG)_2_(NANP)_1_	CGGGGGGGGGGNANP
(NANP)_2_	CGGNANPNANP
(NANP)_6_	CGGNANPNANPNANPNANPNANPNANP
RI short	CGGEDNEKLRKPKH
RI long	CGGDDGNNEDNEKLRKPKHKKLKQPADGN
ADGN long	ADGNPDPNANPNVDPGGC
(NVDP)_6_	CGGNVDPNVDPNVDPNVDPNVDPNVDP

### ELISAs: standard curve, affinity and subclass

Nunc Maxisorp 96-well plates (Sigma) were coated with antigen (50 µl, 1 µg/mL in PBS) and incubated overnight at room temperature (RT). Plates were washed 6 times with PBS/0.05% Tween (PBS/T) (Sigma) and blocked for 1 h with 10% skimmed milk (Sigma) in PBS/T (100 µL/well). Microvette serum tubes (Sarstedt) were used to collect blood from the tail veins of mice and serum obtained by centrifugation (45,000*g*, 10 min). Sera was typically diluted at 1:500 post-prime, 1:1000 post-second shot and 1:2000 post-third shot and applied to plates in triplicate after blocking (2 h RT incubation). Standard curves were prepared on each plate against an antigen of interest by serial dilution of standard sera obtained by a cardiac bleed from mice vaccinated with the specific antigen being tested in ELISA. Plates were washed as before and goat anti-mouse whole IgG alkaline phosphatase conjugate (Sigma) applied (50 µl/well, 1:5000 in PBS/T, 1 h RT). Plates were washed as before and 1 mg/mL pNPP (Sigma) in diethanolamine buffer (Pierce) applied to the plates (100 µl/well) and allowed to develop with readings on a BioTech Microplate Reader taken at 14 min and 1 h at 405 nm. Titres were expressed as arbitrary ELISA units (EU) relative to a standard curve.

To determine the avidity index, a replicate ELISA was performed identical to and simultaneously with the standard curve ELISA, except that after 2 h incubation with diluted sera, 100 µL 7 M urea (Sigma) was applied to each well for 10 min (excluding the standard curve). Plates were then washed and the ELISA completed as before. The avidity index is the ratio of urea-treated to untreated ELISA units^35^.

### Statistical tests used

GraphPad Prism (MacOS v6) and Microsoft Excel were used for all statistical analyses performed. Student’s *t* test and ANOVA with Bonferroni’s multiple comparisons test were used on parametric data comparing two or more groups respectively. Log-rank (Mantel-Cox) tests were used to determine significant differences between survival curves.

### Statistical definition of challenge outcomes

Three sporozoite challenge outcomes used in this paper are defined as follows. Sterile protection, or “protection”, represents a case where a mouse has no detectable blood-stage parasitaemia by fifteen days post-infection. A mouse is classified as “delayed” if the time to reach 1% blood-stage parasitaemia in that mouse is greater than the meantime to reach 1% blood-stage parasitaemia of naïve or control mice in that experiment plus two standard deviations. A mouse is “not delayed” if the time to reach 1% blood-stage parasitaemia is less than two standard deviations above the mean of naïve or control mice.

### Reporting summary

Further information on experimental design is available in the Nature Research Reporting Summary linked to this paper.

## Supplementary information

Reporting Summary

## Data Availability

The data supporting the findings of the study are available from the corresponding author upon request.
