# Supplementary material for: A VLP for validation of the Plasmodium falciparum circumsporozoite protein junctional epitope for vaccine development
Source: NPJ Vaccines. 2021 Apr 1;6:46. doi: 10.1038/s41541-021-00302-x (PMC8016880; doi:10.1038/s41541-021-00302-x)
Supplement: Supplementary file 1 — Reporting Summary [file 41541_2021_302_MOESM1_ESM.pdf]

nature research

Corresponding author(s): Arturo Reyes-Sandoval

Last updated by author(s): 26-01-2021

Reporting Summary

Nature Research wishes to improve the reproducibility of the work that we publish. This form provides structure for consistency and transparency in reporting. For further information on Nature Research policies, see our Editorial Policies and the Editorial Policy Checklist.

Statistics

For all statistical analyses, confirm that the following items are present in the figure legend, table legend, main text, or Methods section.

n/a	Confirmed	
☐	☒	The exact sample size (*n*) for each experimental group/condition, given as a discrete number and unit of measurement
☐	☒	A statement on whether measurements were taken from distinct samples or whether the same sample was measured repeatedly
☐	☒	The statistical test(s) used AND whether they are one- or two-sided<br>*Only common tests should be described solely by name; describe more complex techniques in the Methods section.*
☐	☒	A description of all covariates tested
☐	☒	A description of any assumptions or corrections, such as tests of normality and adjustment for multiple comparisons
☐	☒	A full description of the statistical parameters including central tendency (e.g. means) or other basic estimates (e.g. regression coefficient) AND variation (e.g. standard deviation) or associated estimates of uncertainty (e.g. confidence intervals)
☒	☐	For null hypothesis testing, the test statistic (e.g. *F*, *t*, *r*) with confidence intervals, effect sizes, degrees of freedom and *P* value noted<br>*Give P values as exact values whenever suitable.*
☒	☐	For Bayesian analysis, information on the choice of priors and Markov chain Monte Carlo settings
☒	☐	For hierarchical and complex designs, identification of the appropriate level for tests and full reporting of outcomes
☒	☐	Estimates of effect sizes (e.g. Cohen's *d*, Pearson's *r*), indicating how they were calculated

Our web collection on statistics for biologists contains articles on many of the points above.

Software and code

Policy information about availability of computer code

Data collection	N/A
Data analysis	PRISM 8

For manuscripts utilizing custom algorithms or software that are central to the research but not yet described in published literature, software must be made available to editors and reviewers. We strongly encourage code deposition in a community repository (e.g. GitHub). See the Nature Research guidelines for submitting code & software for further information.

Data

Policy information about availability of data

All manuscripts must include a data availability statement. This statement should provide the following information, where applicable:

- Accession codes, unique identifiers, or web links for publicly available datasets
- A list of figures that have associated raw data
- A description of any restrictions on data availability

Thedatasetsgeneratedduringand/oranalysedduringthecurrentstudyareavailablefromthecorrespondingauthoronreasonablerequest.

Field-specific reporting

Please select the one below that is the best fit for your research. If you are not sure, read the appropriate sections before making your selection.

- [x] Life sciences
- [ ] Behavioural & social sciences
- [ ] Ecological, evolutionary & environmental sciences

For a reference copy of the document with all sections, see nature.com/documents/nr-reporting-summary-flat.pdf

Life sciences study design

All studies must disclose on these points even when the disclosure is negative.

Sample size	A power analysis was performed before an experiment to find the minimum number of mice that could give a statistical difference, which varied depending on the nature of the assay, antibody or T cell response
Data exclusions	Data was excluded in animals with other non-related diseases
Replication	Results in paper were confirmed upon replication of the experiments at least once.
Randomization	Mice were assigned to cages randomly. We use inbred mice for this, thus reducing variability
Blinding	Most experiments were performed by one scientists and were not blinded, being the scientist's particular project. However, we have performed experiments by different scientists and found reproducibility.

Reporting for specific materials, systems and methods

We require information from authors about some types of materials, experimental systems and methods used in many studies. Here, indicate whether each material, system or method listed is relevant to your study. If you are not sure if a list item applies to your research, read the appropriate section before selecting a response.

Materials & experimental systems

n/a	Involved in the study	
[ ]	[x]	Antibodies
[ ]	[x]	Eukaryotic cell lines
[x]	[ ]	Palaeontology and archaeology
[ ]	[x]	Animals and other organisms
[x]	[ ]	Human research participants
[x]	[ ]	Clinical data
[x]	[ ]	Dual use research of concern

Methods

n/a	Involved in the study	
[x]	[ ]	ChIP-seq
[x]	[ ]	Flow cytometry
[x]	[ ]	MRI-based neuroimaging

Antibodies

Antibodies used	Plates were washed as before and goat anti-mouse whole IgG alkaline phosphatase conjugate (Sigma) applied (50 μl/well, 1:5000 in PBS/T, 1 h RT).
Validation	Data is available at Sigma's website: https://www.sigmaaldrich.com/catalog/product/sigma/a9316?lang=en&region=GB&gclid=CjwKCAjwkJj6BRA-EiwA0ZVPVvPdP1tgvjYOIHNkPNUXMSpblsGFCWM38Nr0uIIjPfoEeio-e3oy2RoCudAQAvD_BwE

Eukaryotic cell lines

Policy information about cell lines

Cell line source(s)	ATCC collection
Authentication	Cells were obtained from ATCC
Mycoplasma contamination	Test for Mycoplasma infection was performed.
Commonly misidentified lines (See ICLAC register)	N/A

Animals and other organisms

Policy information about studies involving animals; ARRIVE guidelines recommended for reporting animal research

Laboratory animals	6 week-old female BALB/c (H-2d) mice were used for vaccination experiments, with age-matched controls. TO outbred mice and BALB/c mice were used for parasite maintenance and mosquito feeds. All mice from Harlan/Envigo.
Wild animals	*Provide details on animals observed in or captured in the field; report species, sex and age where possible. Describe how animals were caught and transported and what happened to captive animals after the study (if killed, explain why and describe method; if released, say where and when) OR state that the study did not involve wild animals.*
Field-collected samples	n/a
Ethics oversight	All animals and procedures were used in accordance with the terms of the United Kingdom Home Office Animals Act Project License. The procedures were approved by the University of Oxford Animal Care and Ethical Review Committee (PPL 30/2889 and P9804B4F1).

Note that full information on the approval of the study protocol must also be provided in the manuscript.
